# Compounds with Distinct Targets Present Diverse Antimicrobial and Antibiofilm Efficacy against *Candida albicans* and *Streptococcus mutans,* and Combinations of Compounds Potentiate Their Effect

**DOI:** 10.3390/jof7050340

**Published:** 2021-04-28

**Authors:** Carmélia Isabel Vitorino Lobo, Ana Carolina Urbano de Araújo Lopes, Marlise Inêz Klein

**Affiliations:** Department of Dental Materials and Prosthodontics, School of Dentistry, São Paulo State University (Unesp), Araraquara. Rua Humaitá, 1680, Araraquara, São Paulo 14801-903, Brazil; carmelialobo@gmail.com (C.I.V.L.); carolina.urbano@unesp.br (A.C.U.d.A.L.)

**Keywords:** biofilm, *Candida albicans*, *Streptococcus mutans*, extracellular matrix, antimicrobial agents, antibiofilm agents

## Abstract

*Candida albicans* and *Streptococcus mutans* interact synergistically in biofilms associated with a severe form of dental caries. Their synergism is driven by dietary sucrose. Thus, it is necessary to devise strategies to hinder the development of those biofilms and prevent cavities. Six compounds [*tt*-farnesol (sesquiterpene alcohol that decreases the bacterium acidogenicity and aciduricity and a quorum sensing fungal molecule), myricetin (flavonoid that interferes with *S. mutans* exopolysaccharides production), two 2’-hydroxychalcones and 4’-hydroxychalcone (intermediate metabolites for flavonoids), compound 1771 (inhibitor of lipoteichoic synthase in Gram-positive bacteria)] with targets in both fungus and bacterium and their products were investigated for their antimicrobial and antibiofilm activities against single-species cultures. The compounds and concentrations effective on single-species biofilms were tested alone and combined with or without fluoride to control initial and pre-formed dual-species biofilms. All the selected treatments eliminated both species on initial biofilms. In contrast, some combinations eliminated the bacterium and others the fungus in pre-formed biofilms. The combinations 4’-hydroxychalcone+*tt*-farnesol+myricetin, 4’-hydroxychalcone+*tt*-farnesol+fluoride, and all compounds together with fluoride were effective against both species in pre-formed biofilms. Therefore, combinations of compounds with distinct targets can prevent *C. albicans* and *S. mutans* dual-species biofilm build-up in vitro.

## 1. Introduction

Several human diseases are caused by dysbiotic biofilms, including tooth decay, periodontal diseases, and oral candidiasis [1]. *Candida albicans* is an opportunistic species that, when associated with *Streptococcus mutans,* contributes to forming a complex and organized biofilm that is more tolerant to environmental stresses, including antimicrobial [2]. The interaction between these two species is synergistic in the presence of dietary sucrose and leads to severe dental caries lesions [3]. Therefore, it is necessary to devise strategies to hinder the development of those biofilms.

Within the complex oral microbiota, *S. mutans* is a producer of the extracellular matrix and modulates cariogenic biofilm formation when sucrose from the host’s diet is available [4]. This bacterium is acidogenic and aciduric but not the most numerous species in the mouth, and there are other acidogenic and aciduric microorganisms [5,6]. However, its exoenzymes glucosyltransferases (Gtfs) and fructosyltransferase (Ftf) use sucrose as a substrate for the synthesis of exopolysaccharides (α-glucans and fructans), important components of biofilm construction [4]. Gtfs also adsorb on the surface of other oral microorganisms, converting them into glucan producers [4]. *C. albicans* is one of the microorganisms to which Gtfs binds and form high amounts of exopolysaccharides [7]. This fungus is also acidogenic and aciduric [8], and oral biofilms could serve as reservoirs for it.

The extracellular matrix of *C. albicans* biofilms contains extracellular DNA, β-glucans, mannans, proteins, and lipids [9,10,11,12]. This matrix has been associated with resistance against antifungals [13,14]. Moreover, the biogenesis of this matrix is coordinated extracellularly, reflecting cooperative actions in the biofilm community [14]. Therefore, the production of exopolysaccharides synthesized on surfaces in situ allows adhesion and microbial accumulation [4] and contributes to the construction of the 3D matrix that surrounds and supports cells, forming an environment with acidic niches and limited diffusion [6,15]. Thus, strategies that control the matrix formation could prevent pathogenic biofilms development [13] and perhaps control the amount of *C. albicans* in oral biofilms that could flourish when conditions are favorable. 

The therapeutic modalities for controlling dental biofilm formation are still limited. Chlorhexidine is a broad-spectrum antimicrobial agent that suppresses microorganisms levels in saliva but is not effective against mature biofilms, and its daily and continuous use is not recommended [16]. Fluoride is the mainstay of caries prevention, but its protection against the disease is incomplete [17]. Therefore, an attractive and superior strategy would be to use or include bioactive agents targeting virulence factors and the mechanisms for biofilm development.

Several studies have prioritized finding new antibiofilm agents, including natural substances [18,19,20]. Among the promising agents, *tt*-farnesol (a membrane-targeting sesquiterpenoid) and myricetin (a flavonoid) hinder the development of cariogenic biofilm formed by *S. mutans*. Myricetin inhibits the *gtfs* gene expression and Gtfs activity, thereby hindering the exopolysaccharides synthesis in situ [18,19]. *tt*-farnesol targets the cellular membrane, affecting the tolerance of *S. mutans* to acid stress. Both agents have a moderate biocidal effect [18,19], and their combination with fluoride results in fewer and less severe carious lesions [19]. In addition, *tt*-farnesol is a derivative of the sterol biosynthesis pathway in eukaryotic cells and a *quorum-sensing* molecule of *C. albicans* [21], which keeps the fungus in yeast form. However, it appears not to affect *S. mutans* in concentrations produced when both microorganisms are co-cultivated in biofilms, possible due to the thickness and amount of biomass of these biofilms [2,3]. Additionally, at concentrations above the threshold (i.e., the physiological concentration of the species), *tt*-farnesol can induce apoptosis in planktonic cultures of *C. albicans* cells [22]. Therefore, the antibiofilm effect of *tt*-farnesol and myricetin against *C. albicans* and *S. mutans* biofilms still needs to be investigated [23].

Hydroxychalcones are precursor metabolic intermediates for flavonoids and isoflavonoids. These agents inhibit the streptococcal Gtfs activity [24], impair *S. mutans* survival in planktonic culture [25]; thus, possibly impairing the structure of biofilms. In addition, flavonoids interfere with *C. albicans* cell wall formation, cause disruption of the plasma membrane and mitochondrial dysfunction, affect cell division, protein synthesis, and the efflux-mediated pumping system [26]. Also, synthetic hydroxychalcones were shown to have anti-*Candida* activity [25,27]. Nevertheless, the efficacy of hydroxychalcones on dual-species *S. mutans* and *C. albicans* biofilms is unexplored. 

Also, the interference in the metabolism of lipoteichoic acids (LTA) would affect the development of biofilms by Gram-positive bacteria. The compound 1771 targets LtaS, an LTA synthase enzyme in *S. aureus* [28] and *Enterococcus faecium* [29]. This compound also hinders *S. mutans* biofilm formation [30]. However, the effect of compound 1771 on *C. albicans* is unknown.

Thus, considering the virulence and difficulty of controlling mature biofilms, this study evaluated the antimicrobial and antibiofilm activities of six compounds (*tt*-farnesol, myricetin, two 2′-hydroxychalcones, 4′-hydroxychalcone, and compound 1771) against *C. albicans* and *S. mutans* single- and dual-species settings.

## 2. Materials and Methods

### 2.1. Experimental Design

Antimicrobial activity was evaluated using planktonic cultures of *C. albicans and S. mutans* in microdilution assay to determine the minimum inhibitory concentration (MIC) and minimum fungicidal and bactericidal concentrations (MFC and MBC). Next, single-species biofilms were used to investigate the antibiofilm activities of compounds during initial biofilm formation (24 h) and against pre-formed biofilms (48 h). Finally, promising compounds (and their corresponding concentrations) on both species were combined to analyze the antibiofilm activity on dual-species biofilms formed by *C. albicans* and *S. mutans* on initial biofilm formation (24 h) and pre-formed biofilms (48 h). At that time, fluoride was also added, and groups with and without fluoride were evaluated. All tests were performed using a 96-polystyrene microplate to determine viable microbial population (colony forming units or CFU). At least three independent experiments were performed in triplicate for the antimicrobial and antibiofilm tests (*n* = 9). The data were statistically analyzed according to the factorial design of this study, considering each microplate well as a statistical block. The hypothesis was that elimination or reduction of at least 50% of microbial cells (of both species) from biofilm using the proposed agents and their combinations substantially affect the development of dual-species biofilms.

### 2.2. Test Agents 

An in vitro study with *C. albicans* and *S. mutans* was conducted to investigate the antimicrobial and antibiofilm activity of six compounds: *tt*-farnesol or (E,E)-3,7,11-Trimethyl-2,6,10-dodecatrien-1-ol, trans,trans-3,7,11-Trimethyl-2,6,10-dodecatrien-1-ol (Sigma-Aldrich Co., St Louis, MO, USA; Cat.#46193; 96% purity), myricetin or 3,5,7-trihydroxy-2-(3,4,5-trihydroxyphenyl)-4H-chromen-4-one (AK Scientific, Inc., Union City, CA, USA; Cat.#J10595; 95% purity), three hydroxychalcones [2′-hydroxichalcone or 1-(2-hydroxyphenyl)-3-phenylprop-2-en-1-one (Angene, Hong Kong, China; Cat.#AGN-PC-015IM; 95% purity), 2′-hydroxichalcone or (2E)-1-(2-hydroxyphenyl)-3-phenylprop-2-en-1-one (AK Scientific, Inc.; Cat.#R815; 98% purity), 4′-hydroxichalcone or (2E)-1-(4-hydroxyphenyl)-3-phenylprop-2-en-1-one (AK Scientific, Inc.; Cat.#C135; 98% purity)], and compound 1771 [(5-phenyl-1,3,4-oxadiazol-2-yl)carbamoyl]methyl 2-{naphtho[2,1-b]furan-1-yl}acetate)] (UkrOrgSynthesis Ltd., Kiev, Ukraine; Cat.#PB25353228; purity not available). The compounds were diluted with 84.15% ethanol (EtOH; Sigma-Aldrich; Cat.#E7023) and 15% dimethyl sulfoxide (DMSO; Sigma-Aldrich; Cat.#D8418) to have stock solutions at 15 mg/mL, except for compound 1771 that was used at 2 mg/mL. The concentration for compound 1771 was lower than the other agents because of solubility issues. Also, sodium fluoride was prepared at 5000 ppm (Sigma-Aldrich; Cat.# 71519). These stock solutions were diluted to distinct concentrations for assays. For antimicrobial assays, the agents with stock concentration at 15 mg/mL were tested using concentrations of 1250, 1000, 500, 250, 125, 62.5, 31.25, 15.625 μg/mL. For compound 1771 (stock concentration at 2 mg/mL), the concentrations used were 250, 125, 62.5, 31.25, 15.625, 7.813, 3.906, 1.953, 0.977, 0.488, 0.244 μg/mL.

### 2.3. Microbial Strain and Growth Conditions

*C. albicans* SC5314 and *S. mutans* UA159 (serotype c; ATCC 700610) strains preserved in a freezer −80 °C were thaw, platted on a blood agar plate (5% sheep’s blood; Laborclin, Pinhais, PR, Brazil), and incubated (48 h, 37 °C, 5% CO_2_; Steri-Cult™ Thermo Scientific, Waltham, MA, USA). Next, five colonies of each microorganism were inoculated into a liquid culture medium (2.5% tryptone with 1.5% yeast extract or TY, pH 7.0; Becton, Dickinson and Company (BD), Sparks, MD, USA) containing 1% of glucose (*w*/*v*) (TY+1% glucose) and incubated (16 h, 37 °C, 5% CO_2_). After that, the starter cultures were diluted 1:20 in the same culture medium and grown until mid-log growth phase: *S. mutans* OD_562 nm_ 0.500 (±0.100) and *C. albicans* OD_562nm_ 0.482 (±0.058) (ELISA plate reader, Biochrom Ez, Cambourne, UK). These cultures were diluted in TY+1% glucose for each microorganism inoculum with 2 × 10^6^ colony-forming units per milliliter (CFU/mL) for antimicrobial evaluation. However, the mid-log growth phase cultures were diluted in TY+1% sucrose (*w*/*v*) to yield 2 × 10^6^ CFU/mL for single-species antibiofilm assays.

### 2.4. Antimicrobial Activity

The antimicrobial activity was evaluated by determining the minimum inhibitory concentration (MIC) and minimum fungicidal and bactericidal concentration (MFC and MBC, respectively) using broth microdilution following the Clinical and Laboratory Standards Institute [31,32,33], with some modifications [34]. The compounds were evaluated according their stock concentration ranging from 0 to 1250 μg/mL (when stock concentration 15 mg/mL) and 0 to 250 μg/mL (for compound 1771 with stock concentration at 2 mg/mL). Of note, 0 µg/mL was the vehicle-control. For most of the newest compounds, MIC has been described with some caveats, as when the visual inspection and the optical density are compromised by precipitation, for example [35]. Here, most agents complexed when in contact with the culture medium, making visual inspection subjective and interfering with optical density readings, and most of them did not present a clear dose-dependent effect. Therefore, the abbreviation IC_50_ was defined as the inhibitory concentration of the agent that inhibited the growth of the microorganism by 50% [34], considering microbial growth as the CFU/mL counts. Thus, MIC abbreviation was not employed to state the outcomes.

All selected compounds [*tt*-farnesol, myricetin, 2′-hydroxychalcone (AGN), 2′-hydroxychalcone (R815), 4′-hydroxychalcones (C135), and compound 1771 (1771)] were tested against *S. mutans* planktonic culture. However, only the effective compounds against the bacterium (*tt*-farnesol, myricetin, C135, and compound 1771) were used for *C. albicans* because when both species are together, they form robust biofilms mediated primarily by the exopolysaccharides produced by the bacterium in a rich-sucrose environment [3].

The inoculum culture (100 μL of bacterium or fungus) was transferred to 96-well plates containing TY+1% glucose and distinct concentrations of agents were arranged from the highest to the lowest for a final volume of 200 μL (yielding 1 × 10^6^ CFU/mL for each species). As controls for each experiment, wells containing only culture medium, only inoculum (growth control without treatment), and inoculum plus vehicle (final concentration of 7% EtOH and 1.25% DMSO) were added to rule out any effect of the vehicle on microbial cells. The assembled plates were incubated (24 h, 37 °C, 5% CO_2_), followed visual inspection (turbidity: microbial growth, clear: no growth), and OD_562 nm_ readings (ELISA plate reader). Next, the plates were incubated to homogenize the cultures (5 min, 75 rpm, 37 °C) (Quimis, São Paulo, Brazil). After that, an aliquot from each well was removed for serial dilution in saline solution (0.89% NaCl; Synth, Diadema, SP, Brazil), plating (undiluted and 10^−1^ to 10^−5^), and incubation (48 h, 37 °C, 5% CO_2_) to determine CFU/mL quantification and inhibitory concentration (IC_50_). The MBC and MFC were measured by CFU/mL count and defined as the lowest compound concentration that inhibited microbial growth (or absence of colony growth on agar). However, for some compounds that may target the extracellular matrix production in biofilms, the concentrations that inhibited at least 50% of microbial growth (i.e., 50% of CFU/mL reduction vs. vehicle) were considered promising antimicrobial activity [34,35].

### 2.5. Antibiofilm Activity

This analysis was conducted after determining antimicrobial activity and was performed using single- and dual-species settings and different exposure to compounds: activity against initial biofilm formation (24 h) and pre-formed biofilms (48 h). For initial biofilm formation, the agents were introduced at the time 0 h, and biofilms were evaluated at 24 h of development. For pre-formed biofilms, biofilms were grown for 24 h and then treated, being evaluated at 48 h of growth. Thus, it was evaluated the inhibition of biofilm formation for biofilms at 24 h and the eradication of biofilm growth for 48 h-old biofilms. The measurement was considered effective when the CFU/mL count was reduced by 50% (vs. vehicle) for 24 and 48 h-old biofilms [34,36].

The strains were grown and prepared using the methodology described above. However, the culture medium and inoculum of the experiments were prepared using TY+1% sucrose. The selected compounds and their concentrations were based on antimicrobial data: C135 (from 1250 to 62.5 µg/mL), myricetin (from 1250 to 125 μg/mL), *tt*-farnesol (from 1250 to 31.25 µg/mL), and compound 1771 (from 250 to 1.953 µg/mL). Previous studies evaluated the antibiofilm activity of myricetin, compound 1771, and *tt*-farnesol for *S. mutans* [18,19,30], but here distinct concentrations were tested. Tests with compound 1771 for *C. albicans* and C135 for both species will be presented for the first time.

Initially, single-species biofilm to prevent initial biofilm formation (24 h) were analyzed. On a 96-well plate, 100 µL of final inoculum with 2 × 10^6^ CFU/mL (for both species) were added to each well, containing test concentrations and culture medium (TY+1% sucrose), totalizing 200 µL (1 × 10^6^ CFU/mL). Controls of experiments were also added (wells containing only culture medium, wells containing only the inoculum of the experiment, and wells containing the inoculum plus vehicle or 0 µg/mL). The plate was incubated (24 h, 37 °C, 5% CO_2_). After incubation, visual inspection was performed, followed by orbital incubation (5 min, 75 rpm, 37 °C). The remaining biofilms on the wells were rinsed three times with 200 µL of 0.89% NaCl solution to remove any loose material. Next, these biofilms were scraped with pipet tips five times (5X) with 200 µL of 0.89% NaCl, totalizing 1 mL of biofilm suspension (from each well). This biofilm suspension was placed in a microtube, subjected to serial dilutions (10^−1^ to 10^−5^), which were plated, as were the undiluted suspensions. The plates were incubated (48 h, 37 °C, 5% CO_2_), and the CFU/mL was calculated. Next, the microbial growth inhibition of each concentration was compared to vehicle control.

Subsequently, prevention of build-up pre-formed biofilm (48 h) was evaluated [36]. Here, 96-well plates were assembled using 100 µL final inoculum of *C. albicans* or *S. mutans* (2 × 10^6^ CFU/mL) and 100 µL of TY+1% sucrose (to reach 1 × 10^6^ CFU/mL). The microplate was incubated (24 h, 37 °C, 5% CO_2_) without any treatment or vehicle control. After incubation and biofilm formation, visual inspection was performed, followed by culture medium removal and washing of remaining biofilms (three times with 200 µL 0.89% NaCl solution). Next, fresh culture medium TY+1% sucrose and test concentrations of agents or the vehicle were added. The microplate was incubated again (24 h, 37 °C, 5% CO_2_). After incubation (when biofilms were 48 h-old), the same processing protocol applied for 24 h-old biofilms was conducted until obtaining 1 mL of biofilm suspension. The biofilm suspensions were sonicated (30 s at 7 w, Sonicator QSonica, Q125, Newtown, CT, USA), subjected to serial dilutions (10^−1^ to 10^−5^), and plated. The undiluted suspensions were also plated. The CFU/mL counts were evaluated and compared to vehicle control.

Finally, the antibiofilm activity for dual-species biofilms of *C. albicans* and *S. mutans* was also performed at 24 and 48 h. These analyzes were performed with the same methodology used for single-species biofilms (24 and 48 h). However, the inoculum of the dual-species culture was prepared with 2 × 10^6^ CFU/mL of *S. mutans* and 2 × 10^4^ CFU/mL of *C. albicans* [3] to reach 1 × 10^6^ CFU/mL of *S. mutans* and 1 × 10^4^ CFU/mL of *C. albicans* after adding culture medium or treatments.

The concentrations of agents with better results against each species in the single-species biofilm setting were selected: 125 µg/mL (C135 and *tt*-farnesol), 500 µg/mL (myricetin), and 3.906 µg/mL (1771). Then, compounds with selected concentrations were combined with each other and with or without sodium fluoride (250 ppm) or F totalizing 16 groups. The elected combinations included groups without fluoride (C135, C135+*tt*-farnesol, C135+1771, C135+myricetin, C135+*tt*-farnesol+1771, C135+*tt*-farnesol+myricetin, C135+*tt*-farnesol+myricetin+1771, C135+1771+myricetin, *tt*-farnesol+myricetin, 1771+myricetin, *tt*-farnesol+1771+myricetin) and groups with fluoride (250 ppm) (C135+fluoride, C135+*tt*-farnesol+fluoride, C135+1771+fluoride, C135+myricetin+fluoride, C135+*tt*-farnesol+1771+myricetin+fluoride). The concentration of fluoride was selected based on the commercially available fluoride-based mouth rinse [19,37].

### 2.6. Statistical Analyses

The statistical analyses for CFU/mL values were performed using descriptive and inferential statistics. Normality was evaluated with the Shapiro-Wilk test employing a significance level of 5%. The data were non-parametric; thus, the data were evaluated with Kruskal–Wallis test, followed by Dunn’s post-test, considering α = 0.05 (Prism 9 software, GraphPad Software, Inc. 2021). The microbial growth inhibition of each agent and concentration was compared to vehicle control. In addition, the CFU/mL data were transformed to log_10_ or log to verify the log reduction.

## 3. Results

### 3.1. Antimicrobial Activity

#### 3.1.1. *S. mutans*

Three compounds (AGN, C135, R815) complexed with the culture medium, making the visual inspection analysis inaccurate; turbidity was also present for myricetin (but in minor proportion than for the three aforementioned compounds), and compound 1771 (at concentrations equal of more than 31.25 μg/mL). The observation of culture medium turbidity occurred immediately after adding the compound into the culture media, without microbial inoculation and incubation (controls per each concentration tested). The compound that did not complex with culture medium was *tt*-farnesol, and the absence of visual growth was observed at 31.25 μg/mL, which was also its IC_50_.

Regarding MBC, as per CLSI definition, the absence of colony growth on an agar plate was found for *tt*-farnesol and compound 1771, as 62.5 μg/mL and 250 μg/mL, respectively. Thus, a potential antimicrobial activity was achieved for the compounds when the compound at a specific concentration yielded a 50% reduction of CFU/mL counts compared to the vehicle control (IC_50_), as follows.

**Figure 1:**
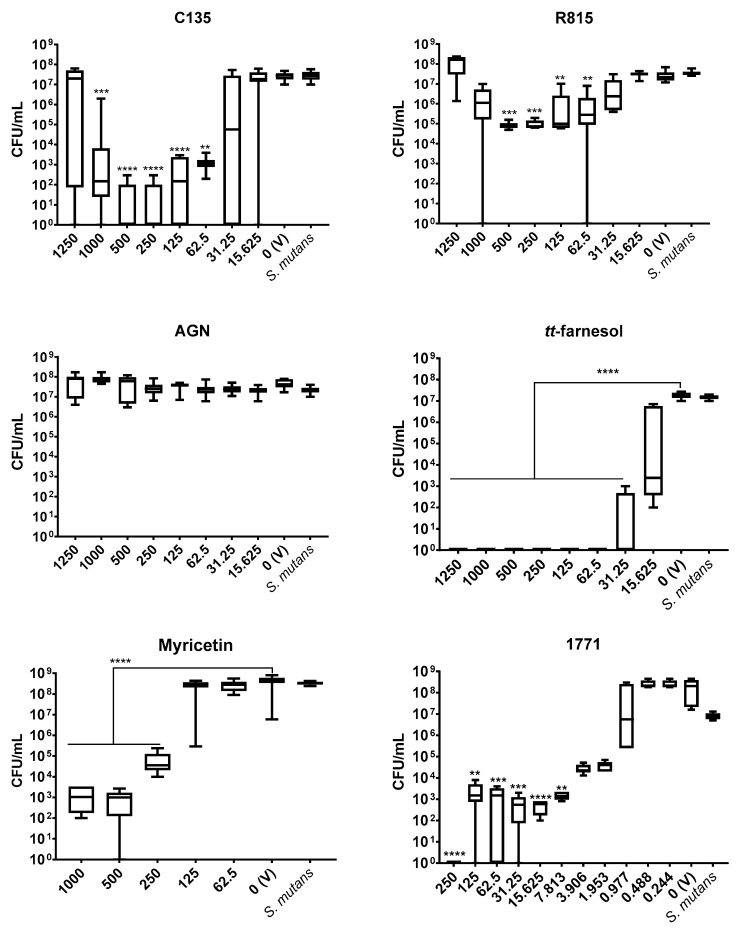
Antimicrobial activity of *S. mutans* using six compounds: C135, R815, AGN, *tt*-farnesol, myricetin and 1771. Data represents median and interquartile ranges (*n* = 9). Statistical differences are represented with ** (*p* < 0.05), *** (*p* < 0.001), and **** (*p* < 0.0001) (Kruskal Wallis, followed by Dunn’s test). The tabulated results are in Table S1.

For C135, the lowest concentration that yielded 50% reduction was 62.5 μg/mL, but the same reduction was observed for 125, 250, and 500 μg/mL (Figure 1). Thus, the antibacterial activity of C135 was not dose-dependent.

There was an absence of expressive effect on growth inhibition for all concentrations of AGN and R815 (vs. vehicle). However, some concentrations of R815 showed statistical differences and some inhibition of growth at 500 and 250 μg/mL (2 and 1 log reduction, respectively), 125 and 62.5 μg/mL (0.5 log reduction). These reductions were not dose-dependent and are not within the cutoff established for an effective compound (Figure 1). Thus, AGN and R815 were not used in the downstream assays.

Three concentrations of myricetin presented statistical differences compared to the vehicle (250, 500, and 1000 μg/mL) but were not dose-dependent. However, a better effect was obtained for 500 μg/mL, which was considered the IC_50_.

For compound 1771, the IC_50_ was 7.813 μg/mL; higher concentrations also demonstrated significative statistical differences (at least 4 log reduction vs. vehicle) and, as mentioned above, 250 μg/mL rendered absence of CFU/mL quantification on agar (MBC).

#### 3.1.2. *C. albicans*

The antimicrobial activity of *C. albicans* was analyzed using four agents (C135, *tt*-farnesol, myricetin, and compound 1771) that were selected based on the effective antimicrobial activity founded for *S. mutans*. Among them, only *tt*-farnesol did not complex with the culture medium; the absence of visual growth was observed at 125 μg/mL, which was also its IC_50_.

**Figure 2:**
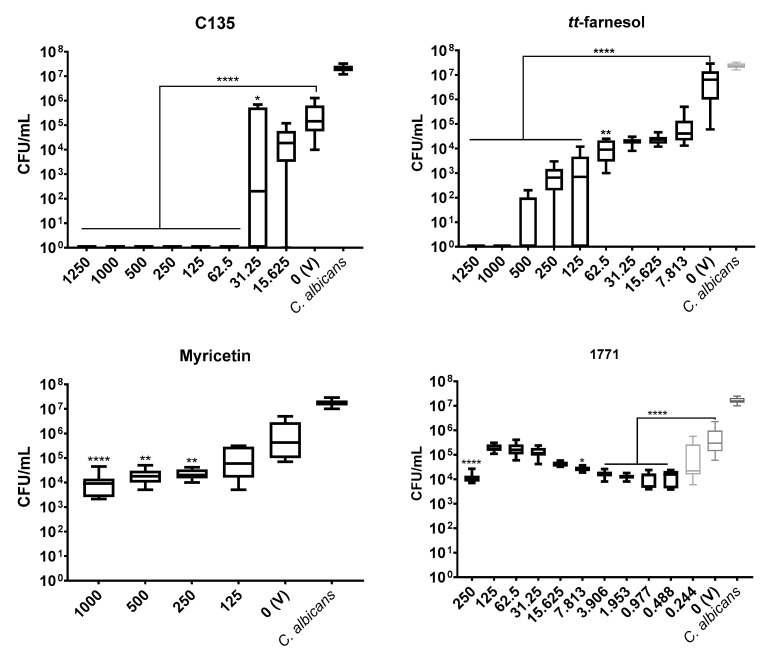
Antimicrobial activity of *C. albicans* with compounds: C135, *tt*-farnesol, myricetin and 1771. Data represents median and interquartile ranges (*n* = 9). Statistical differences are represented with * (C135 *p* = 0.048; 1771 *p* = 0.013), ** (*p* < 0.05) and **** (*p* < 0.0001) (Kruskal Wallis, followed by Dunn’s test). The tabulated results are in Table S2.

C135 and *tt*-farnesol presented a dose-dependent effect on viable fungal growth. C135 was the most effective in inhibiting fungal viability (Figure 2). All its concentrations above 31.25 μg/mL hindered colony growth on agar plates; thus, its IC_50_ and MFC were determined as 62.5 μg/mL (absence of colony growth on agar plates). The MFC for *tt*-farnesol was 1000 μg/mL (Figure 2). Both myricetin and compound 1771 did not demonstrate significant antimicrobial activities as per the cutoff of 50% colony growth reduction (IC_50_), although statistical differences were observed, as depicted in Figure 2.

### 3.2. Antibiofilm Activity

#### 3.2.1. Single-Species *S. mutans* Biofilm

**Figure 3:**
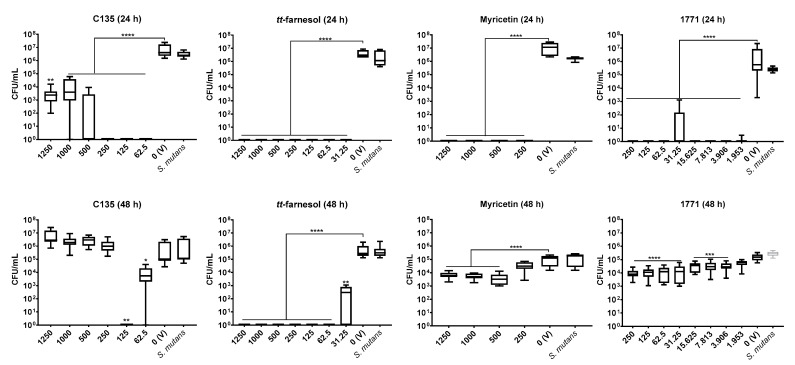
Antibiofilm activity of *S. mutans* with compounds: C135, *tt*-farnesol, myricetin, and 1771. On the first line are presented data of 24 h biofilm (initial biofilm formation); and right below are the data of pre-formed biofilms (48 h). The data represents median and interquartile ranges (*n* = 9). Statistical differences are represented with * (*p* = 0.026), ** (*p* < 0.05), *** (*p* < 0.001), and **** (*p* < 0.0001) (Kruskal Wallis, followed by Dunn’s test). The tabulated results are in Table S3.

On 24 h biofilm (initial biofilm formation)*,* all concentrations of tested compounds (C135, myricetin, *tt*-farnesol, and compound 1771) demonstrated antibiofilm activity, specially myricetin and *tt*-farnesol concentrations that eliminated bacterial growth (Figure 3). C135 eliminated the bacterium at 62.5, 125, and 250 μg/mL, but not at higher concentrations. Also, all concentrations of compound 1771 reduced about 5 logs of bacterium growth (vs. vehicle).

However, on *S. mutans* pre-formed biofilms (48 h) a greater inhibitory effect was achieved with *tt*-farnesol; where concentrations of 62.5 μg/mL and higher eliminated the bacterium. For C135 the best concentration was 125 μg/mL with 5 logs reduction (vs. vehicle). In contrast, a lower inhibitory activity was observed for myricetin and compound 1771 (although they presented statistical differences, the reduction was about 1 log vs. vehicle) (Figure 3).

#### 3.2.2. Single-Species *C. albicans* Biofilm

**Figure 4:**
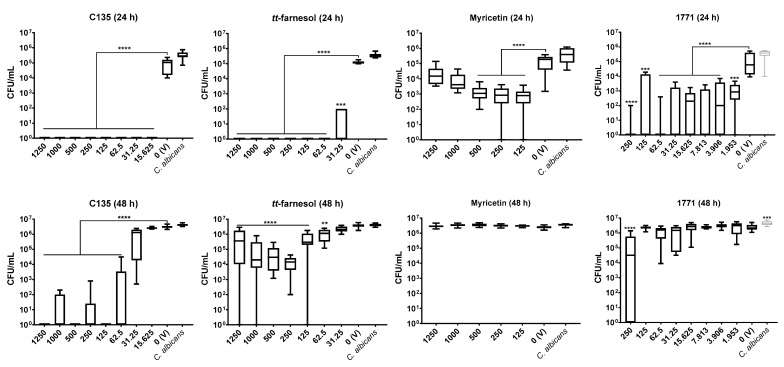
Antibiofilm activity of *C. albicans* with compounds: C135, *tt*-farnesol, myricetin, and 1771. On the first line are presented data of 24 h biofilm (initial biofilm formation); and right below are the data of pre-formed biofilms (48 h). The data represents median and interquartile ranges (*n* = 9). Statistical differences are represented with ** (*p* = 0.002), *** (*p* ≤ 0.0003), and **** (*p* ≤ 0.0001) (Kruskal Wallis, followed by Dunn’s test). The tabulated results are in Table S4.

The best antibiofilm activity for *C. albicans* 24 h biofilm was observed for C135 and *tt*-farnesol; both eliminated the fungus, except at 31.25 μg/mL of *tt*-farnesol that reduced 4 logs (vs. vehicle). Compound 1771 reduced 3 logs from 3.906 μg/mL to higher concentrations. However, the lowest antibiofilm effect was observed for myricetin with about 1 log reduction (vs. vehicle) in all tested concentrations, although statistical differences were observed (Figure 4).

The antibiofilm activity against *C. albicans* pre-formed biofilm (48 h) was achieved effectively only by C135. C135 eliminated the fungus at 125, 500, and 1250 μg/mL; it also decreases CFU/mL counts by 4 logs (62.5 μg/mL) and 5 logs (250 and 1000 μg/mL) (vs. vehicle). A weak activity was observed using *tt*-farnesol, where concentrations above 125 μg/mL presented about 2 log reduction (vs. vehicle). However, no effect was observed using myricetin and compound 1771 (except 1771 at 250 μg/mL with 2 logs reduction vs. vehicle) (Figure 4).

#### 3.2.3. Dual-Species *C. albicans* and *S. mutans* Biofilms.

Based on the previously presented data, compounds and their most effective concentrations were selected for combinations of compounds tested on dual-species *C. albicans* and *S. mutans* biofilms (see data summarized in Table 1). The selected concentrations were: 125 μg/mL for C135 and *tt*-farnesol, 500 μg/mL for myricetin, and 3.906 μg/mL for compound 1771. In addition, C135 was used alone or combined with the other agents with and without sodium fluoride because it was the most effective agent against the fungus growth.

**Figure 5:**
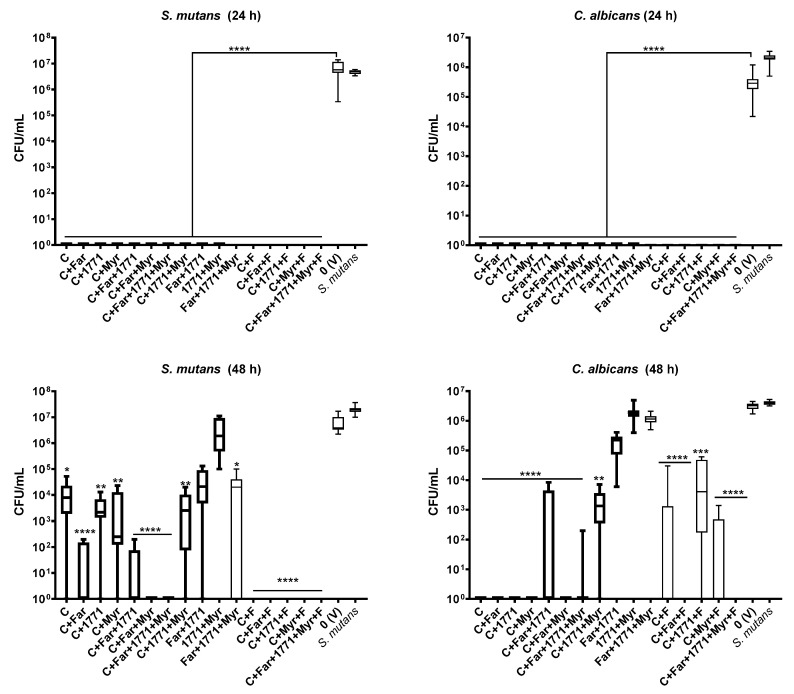
Antibiofilm activity of dual-species *S. mutans* and *C. albicans* biofilms with combined compounds (with and without sodium fluoride): C135 (C), *tt*-farnesol (Far), myricetin (Myr), 1771, and sodium fluoride (F). The top graphs presented data of 24 h biofilm (initial biofilm formation). The bottom graphs depict data of pre-formed biofilms (48 h). The data represents median and interquartile ranges (*n* = 9). Statistical differences are represented with * (*p* = 0.04), ** (*p* ≤ 0.002), *** (*p* = 0.0007), and **** (*p* ≤ 0.0001) (Kruskal Wallis, followed by Dunn’s test). The tabulated results are in Table S5.

For 24 h-old biofilms (initial biofilm formation), all 16 formulations were effective, impeding the growth of both species (bacterium and fungus) (Figure 5). However, the microbial growth inhibition of pre-formed (48 h-old biofilms) was different between treatments and species (Figure 5). Among the 16 formulations tested, three inhibited the growth of both species completely: C135+*tt*-farnesol+myricetin, C135+*tt*-farnesol+fluoride, and C135+*tt*-farnesol+1771+myricetin+fluoride (all compounds combined with fluoride). Furthermore, considering the total inhibition of bacterial growth in the dual-species setting, four formulations were effective (C135+*tt*-farnesol+1771+myricetin, C135+fluoride, C135+1771+fluoride, and C135+myricetin+fluoride). Considering the total inhibition of fungal growth in the dual-species setting, four treatments were effective (C135, C135+*tt*-farnesol, C135+1771, and C135+myricetin). Also, some formulations reduced at least 50% CFU/mL (vs. vehicle) of *S. mutans* (C135+*tt*-farnesol, C135+*tt*-farnesol+1771), or *C. albicans* (C135+*tt*-farnesol+1771+myricetin and C135+myricetin+fluoride).

## 4. Discussion

Several strategies can be used to control biofilms to prevent oral diseases. The classical strategies for oral biofilm control include brushing/flossing (mechanical removal of biofilms) and restricting dietary sugar intake (mainly frequency) to prevent biofilm build-up. Both diet restriction and brushing/flossing require behavioral compliance, which can be challenging. In addition, fluoride is used to avoid teeth demineralization and promote remineralization as part of oral hygiene products (toothpaste and mouthwashes) and/or supplied in tap drinking water. However, they may not be appropriate to all individuals, such as those without adequate dexterity (e.g., young children, elderly, people with disabilities, people in ICUs), which may require supervision for brushing/flossing and aids to weaken the overall biofilm structure to facilitate its mechanical removal, or even substances to enhance biofilm control.

Single targets for biofilm prevention and antimicrobial control can be difficult and limit the treatment options. Thus, combining agents with distinct targets can be an effective approach to access different sites in biofilms, which present complex biological traits and protected niches [1,19]. Therefore, this study evaluated the antimicrobial and antibiofilm activities of six compounds [*tt*-farnesol, myricetin, two 2′-hydroxychalcones (AGN and R815), 4′-hydroxychalcone (C135), and compound 1771] with different targets against *C. albicans* and *S. mutans* single- and dual-species settings.

Of note, an antimicrobial substance/molecule may not be an antibiofilm agent, and a compound with antibiofilm activity may not be an antimicrobial per se (e.g., molecules that affect extracellular enzymes responsible for the extracellular matrix construction). This scenario is depicted by the findings in both antimicrobial and antibiofilm assays performed as some agents were effective against the microorganisms in planktonic cultures and were not in the biofilms’ settings. Also, the effect of compounds was mostly non-dose-dependent for both *C. albicans* and *S. mutans*.

The antimicrobial outcome for *S. mutans* showed the lowest IC_50_ value for compound 1771 (7.813 μg/mL), followed by *tt*-farnesol (31.25 μg/mL), C135 (62.5 μg/mL), while the highest value was observed for myricetin (500 μg/mL). However, IC_50_ values for *C. albicans* were C135 (62.5 μg/mL) and *tt*-farnesol (125 μg/mL). Furthermore, *tt*-farnesol eliminated CFU/mL count of both species reaching a MBC (62.5 μg/mL) and a MFC (1000 μg/mL). The compound 1771 presented MBC (250 μg/mL) and C135 reached MFC (62.5 μg/mL). The findings for C135 for both species and 1771 for *C. albicans* are presented for the first time here. Thus, C135 presented a promising antimicrobial effect for both species, and compound 1771 did not inhibit *C. albicans* growth.

A previous study with *S. mutans* using different concentrations of compound 1771 did not eliminate the bacterium [30], but the total elimination of CFU/mL count was observed here using the greatest concentration. In the same study [30], the antimicrobial activity of myricetin for *S. mutans* was at a lower concentration than the one found here. The antimicrobial effect of *tt*-farnesol on both species corroborates previous findings [18,22]. Among the three chalcones tested, the antimicrobial activity was better for C135, a 4′-hydroxychalcone. The other two 2′-hydroxychalcones (AGN and R815) did not present antimicrobial effect for *S. mutans*. These results can be explained by the differences between the chemical structure of the selected hydroxychalcones, suggesting that the presence of hydroxyl groups on the ring of the 4′-hydroxychalcone scaffold is crucial for the growth inhibition [24,38,39].

The presence of an extracellular matrix is essential for the existence of biofilm and the complete expression of virulence by microbial pathogens and pathobionts, hindering the action of antimicrobial agents and preventing their access to microbial cells [40]. Sucrose can modulate microbial synergism and ecology of the oral microbiota because its hexoses (glucose and fructose) are used for exopolysaccharides and organic acid production that influences the structure and composition of dental biofilms [1,6]. Cariogenic biofilms promote interactions and mechanisms that control dysbiosis [1,6] as observed on dual-species biofilms of *S. mutans* and *C. albicans* in vitro [2,3]. Therefore, it is important to understand the mechanisms of possible antibiofilm compounds.

Myricetin and some hydroxychalcones inhibit *S. mutans* F-ATPase activity (acid tolerance mechanism) [19], glycolysis (organic acid production or acidogenicity) [19], and synthesis of extracellular matrix glucans (by interfering with *gtfs* gene expression and Gtfs activity) [19,24]. The deficit in glucan production can compromise the integrity and 3D structure biofilms [4], facilitating their disruption. These findings can explain the antibiofilm effect of myricetin on *S. mutans* initial biofilms (24 h) and the greatest potential of C135 (4′-hydroxychalcone) on initial (24 h) and mature *S. mutans* biofilm (48 h). The weaker inhibition of myricetin on pre-formed biofilms (48 h—biofilm eradication) can be promoted by the presence of pre-formed microcolonies and their 3D extracellular matrix.

*tt*-farnesol eliminated the bacterium in 24- and 48 h-old single-species biofilms (at 62.5 μg/mL and higher concentrations). The antimicrobial and antibiofilm effect of this compound can be related to the targets on *S. mutans* cytoplasmatic membrane, altering its proton permeability, decreasing its tolerance to acid stress [18]. Compound 1771 also had a promising antibiofilm effect for the bacterium, especially on initial biofilm (24 h-old single-species biofilms). This compound inhibits the LTA synthesis [28], interfering with the cell wall composition, making the cytoplasmatic membrane an easy target for environmental stresses. Also, LTA from the cell wall are released in the matrix and interact with exopolysaccharides during the development and maturation of biofilms [41]; hence, interfering with LTA metabolism can impair cell wall and extracellular matrix composition. Thus, *tt*-farnesol and compound 1771 hindered *S. mutans* biofilms by promoting antimicrobial and antibiofilm activities.

Hydroxychalcones inhibit the cell wall formation of *C. albicans* cells [42,43], while *tt*-farnesol keeps the fungus in its yeast form [21]. However, it is unknown whether myricetin inhibits *C. albicans* biofilm formation or its extracellular matrix development or whether compound 1771 could target this fungus or its metabolism. The effect on the extracellular matrix components and construction could make the fungal cells more susceptible to antimicrobial agents. Here, single-species *C. albicans* biofilms were not greatly affected by myricetin. However, the 4′-hydroxychalcone C135 presented a promising effect in the initial (24 h) and mature (48 h) fungal biofilms. Also, *tt*-farnesol and 1771 were effective on initial fungal biofilm, but only *tt*-farnesol eliminated the fungus (at 62.5 μg/mL and higher concentrations). In addition, *tt*-farnesol had a weak effect, while 1771 had practically no effect on fungal growth on pre-formed biofilms. Thus, eradication of *C. albicans* biofilm (48 h) was achieved only with C135.

Previous studies with *C. albicans* biofilms demonstrate that chalcones inhibited enzymes involved in resistance pathways [42]. Thus, the effect of C135 on *C. albicans* biofilms can be related to targets on resistance pathways; nevertheless, this hypothesis must be better explained. In addition, as observed on the data above from antimicrobial activity of *tt*-farnesol, this compound can inhibit fungal growth, induce apoptosis in *C. albicans* cells, and inhibit the fungal hyphae [21,22]. As described for initial biofilms (24 h), the treatment was applied at 0 h, so it may be that the effect of *tt*-farnesol (preventing filamentous morphology) supports the inhibition of fungal growth in 24 h while hampered its effect on pre-formed biofilm (48 h). Therefore, combine compounds could improve the effect in mature biofilms. This hypothesis is confirmed by the potentiated effect on *C. albicans* cells in dual-species biofilm (48 h) when C135 and *tt*-farnesol were combined, suggesting inhibition of the resistance mechanisms when both compounds are present.

Altogether, the data from antimicrobial and single-species biofilms assays enabled the selection of specific concentrations of the four compounds (C135, myricetin, *tt*-farnesol, and compound 1771) that were combined, with or without sodium fluoride, to assess the antibiofilm activity of formulations against dual-species *S. mutans* and *C. albicans* biofilm. All formulations without fluoride [(C135, C135+*tt*-farnesol, C135+1771, C135+myricetin, C135+*tt*-farnesol+1771, C135+*tt*-farnesol+myricetin, C135+*tt*-farnesol+1771+myricetin, C135+1771+myricetin, *tt*-farnesol+1771, 1771+myricetin, *tt*-farnesol+1771+myricetin)] and with fluoride [(C135+fluoride, C135+*tt*-farnesol+fluoride, C135+1771+fluoride, C135+J10595+fluoride, C135+*tt*-farnesol+1771+myricetin+fluoride completely inhibited the initial biofilm formation (24 h). These findings showed that there might be a potential synergism between the compounds and a greater effect when they are combined and applied since the beginning of biofilm formation and during the 24 h of dual-species biofilm development. Part of the effect can be because of the antimicrobial effect per se, as microbial cells were exposed to formulations in their free form before adhesion to the surface. Also, the effect on extracellular matrix formation can not be ruled out.

In contrast, dual-species biofilm eradication (48 h) in which both species did not grow occurred for three formulations: C135+*tt*-farnesol+myricetin, C135+*tt*-farnesol+fluoride, and C135+*tt*-farnesol+1771+myricetin+fluoride. Four formulations only eradicated the bacterial growth (C135+*tt*-farnesol+1771+myricetin, C135+fluoride, C135+1771+fluoride, and C135+myricetin+fluoride), while other four formulations eradicated fungal growth (C135, C135+*tt*-farnesol, C135+1771, and C135+myricetin). Of note, the formulations with fluoride exhibited a greater antibiofilm activity (mainly for the bacterium), reinforcing the importance of its inclusion in strategies to prevent and control cariogenic biofilms [18]; fewer and less severe carious lesions were observed using combined treatments and fluoride on a rodent model of dental caries [19]. Fluoride can interfere with microbial metabolism, especially on glycolytic enzymes and proton gradient dissipation on the cell cytoplasmatic membrane (when the extracellular pH is higher than the intracellular pH) [44]. This effect can hamper cell growth. Nevertheless, C135 alone or combined with other agents (even those without pronounced effect on singles-species 48 h-old biofilms) prevented fungal and bacterial growth in dual-species biofilms, making it a promising agent.

The present findings provided insights about: (i) compounds as inhibitors of biofilm formation of single-species biofilm (24 h); (ii) compounds that can eradicate pre-formed biofilm (48 h); and (iii) formulations with combined compounds for biofilm inhibition (24 h) and eradication (48 h) of both species in dual-species biofilms. C135 is a novel compound with possible distinct targets alone or in combination with other agents. The formulations that combined agents with distinct targets prevented *C. albicans* and *S. mutans* dual-species biofilm build-up in vitro. The formulation C135+*tt*-farnesol with or without fluoride may represent a potential alternative approach that deserves further investigation, including cytotoxicity to host [30,45]. Therefore, the outcomes of this study could be applied to future studies using the compound alone or combined as an adjuvant strategy to control oral biofilms using shorter exposure times, as mouthwashes.

## Figures and Tables

**Table 1:** Summary of antimicrobial and antibiofilm activity on single-species cultures for selection of compounds concentrations (μg/mL) to test against dual-species biofilms.

Compound	Antimicrobial Activity				Antibiofilm Activity (Single-Species)			
	*S. mutans*		*C. albicans*		*S. mutans*		*C. albicans*	
	IC_50_	MBC	IC_50_	MFC	24 h	48 h	24 h	48 h
C135	62.5	-	62.5	62.5	62.5	125	15.625	125
*tt*-farnesol	31.25	62.5	125	1000	31.25	62.5	62.5	125 *
Myricetin	500	-	-	-	250	500 *	500 *	-
1771	7.813	250	-	-	3.906	3.906 *	3.906 *	250 *

* represent selected concentrations that did not reduce 50% of CFU/mL but had a significative statistical reduction vs. vehicle between all tested concentrations. IC_50_: the inhibitory concentration of the agent that inhibited the growth of the microorganism by 50%, considering microbial growth as the CFU/mL counts. MBC: minimum bactericidal concentration. MFC: minimum fungicidal concentration. 24 h: 24 h-old biofilms. 48 h: 48 h-old biofilms.

## Data Availability

The data presented in this study are available as supplementary material. Additional data are available on request from the corresponding author.

## Supplementary Materials

The following are available online at https://www.mdpi.com/article/10.3390/jof7050340/s1. Table S1: Antimicrobial activity of *S. mutans* using six compounds: C135, R815, AGN, *tt*-farnesol, myricetin, and 1771. Data shown in Figure 1 as CFU/mL. Table S2: Antimicrobial activity of *C. albicans* with compounds: C135, *tt*-farnesol, myricetin, and 1771. Data shown in Figure 2 as CFU/mL. Table S3: Antibiofilm activity of *S. mutans* with compounds: C135, *tt*-farnesol, myricetin, and 1771 of 24 h biofilm (initial biofilm formation) and pre-formed biofilms (48 h). Data shown in Figure 3 as CFU/mL. Table S4: Antibiofilm activity of *C. albicans* with compounds: C135, *tt*-farnesol, myricetin, and 1771 of 24 h biofilm (initial biofilm formation) and pre-formed biofilms (48 h). Data shown in Figure 4 as CFU/mL. Table S5. Antibiofilm activity of dual-species *S. mutans* and *C. albicans* biofilms with combined compounds (with and without sodium fluoride): C135 (C), *tt*-farnesol (Far), myricetin (Myr), 1771, and sodium fluoride (F). Data shown in Figure 5 as CFU/mL.
