# Supplementary material for: Compounds with Distinct Targets Present Diverse Antimicrobial and Antibiofilm Efficacy against Candida albicans and Streptococcus mutans, and Combinations of Compounds Potentiate Their Effect
Source: J Fungi (Basel). 2021 Apr 28;7(5):340. doi: 10.3390/jof7050340 (PMC8146248; doi:10.3390/jof7050340)
Supplement: Supplementary file 1 [file jof-07-00340-s001.zip › jof-1187236-supplementary.pdf]

Compounds with distinct targets present diverse antimicrobial and antibiofilm efficacy against *Candida albicans* and *Streptococcus mutans,* and combinations of compounds potentiate their effect

Supplementary Information

Tables with tabulated results are presented for Figures depicting graphs in the main document.

Table S1. Antimicrobial activity of *S. mutans* using six compounds: C135, R815, AGN, *tt*-farnesol, myricetin, and 1771. Data shown in Figure 1 as CFU/mL.

C135

	1250	1000	500	250	125	62.5	31.25	15.625	0 (V)	*S. mutans*
Minimum	0.00E+00	0.00E+00	0.00E+00	0.00E+00	0.00E+00	2.00E+02	0.00E+00	0.00E+00	1.00E+07	1.00E+07
25% Percentile	7.50E+01	2.50E+01	0.00E+00	0.00E+00	0.00E+00	7.75E+02	0.00E+00	1.38E+07	1.83E+07	1.78E+07
Median	2.00E+07	1.50E+02	0.00E+00	0.00E+00	1.50E+02	1.15E+03	5.75E+04	1.85E+07	2.85E+07	2.75E+07
75% Percentile	5.05E+07	6.53E+03	1.00E+02	1.00E+02	2.40E+03	1.70E+03	2.75E+07	4.00E+07	3.60E+07	4.23E+07
Maximum	6.40E+07	2.00E+06	3.00E+02	3.00E+02	3.00E+03	4.00E+03	5.30E+07	6.20E+07	4.80E+07	5.80E+07
Range	6.40E+07	2.00E+06	3.00E+02	3.00E+02	3.00E+03	3.80E+03	5.30E+07	6.20E+07	3.80E+07	4.80E+07

R815

	1250	1000	500	250	125	62.5	31.25	15.625	0 (V)	S. mutans
Minimum	1.40E+06	0.00E+00	5.00E+04	6.40E+04	5.90E+04	0.00E+00	4.00E+05	1.40E+07	1.20E+07	2.60E+07
25% Percentile	3.00E+07	1.68E+05	6.25E+04	6.50E+04	6.28E+04	9.00E+04	4.88E+05	2.73E+07	1.50E+07	2.85E+07
Median	1.65E+08	1.14E+06	8.50E+04	7.20E+04	1.00E+05	2.85E+05	2.40E+06	3.20E+07	2.20E+07	3.60E+07
75% Percentile	2.23E+08	5.20E+06	1.08E+05	1.45E+05	2.59E+06	1.98E+06	1.55E+07	3.68E+07	3.78E+07	4.08E+07
Maximum	2.40E+08	1.00E+07	1.60E+05	2.00E+05	1.03E+07	8.00E+06	3.10E+07	4.40E+07	6.90E+07	6.00E+07
Range	2.39E+08	1.00E+07	1.10E+05	1.36E+05	1.02E+07	8.00E+06	3.06E+07	3.00E+07	5.70E+07	3.40E+07

AGN

	1250	1000	500	250	125	62.5	31.25	15.625	0 (V)	S. mutans
Minimum	4.00E+06	4.50E+07	3.00E+06	6.40E+06	7.00E+06	6.00E+06	1.10E+07	6.00E+06	1.70E+07	1.00E+07
25% Percentile	8.25E+06	5.35E+07	4.55E+06	1.53E+07	3.50E+07	1.53E+07	1.70E+07	1.63E+07	3.05E+07	1.70E+07
Median	8.10E+07	7.50E+07	6.15E+07	2.55E+07	3.95E+07	2.70E+07	2.40E+07	1.95E+07	4.20E+07	2.05E+07
75% Percentile	1.02E+08	9.88E+07	9.65E+07	4.03E+07	4.25E+07	2.78E+07	3.33E+07	2.73E+07	7.63E+07	2.83E+07
Maximum	1.70E+08	1.70E+08	1.21E+08	8.40E+07	5.00E+07	7.40E+07	5.10E+07	3.90E+07	8.00E+07	4.00E+07
Range	1.66E+08	1.25E+08	1.18E+08	7.76E+07	4.30E+07	6.80E+07	4.00E+07	3.30E+07	6.30E+07	3.00E+07

tt-farnesol

	1250	1000	500	250	125	62.5	31.25	15.625	0 (V)	*S. mutans*
Minimum	0.00E+00	0.00E+00	0.00E+00	0.00E+00	0.00E+00	0.00E+00	0.00E+00	1.00E+02	1.00E+07	1.00E+07
25% Percentile	0.00E+00	0.00E+00	0.00E+00	0.00E+00	0.00E+00	0.00E+00	0.00E+00	3.75E+02	1.40E+07	1.40E+07
Median	0.00E+00	0.00E+00	0.00E+00	0.00E+00	0.00E+00	0.00E+00	0.00E+00	2.45E+03	1.80E+07	1.50E+07
75% Percentile	0.00E+00	0.00E+00	0.00E+00	0.00E+00	0.00E+00	0.00E+00	4.75E+02	5.70E+06	2.40E+07	1.60E+07
Maximum	0.00E+00	0.00E+00	0.00E+00	0.00E+00	0.00E+00	0.00E+00	1.00E+03	7.10E+06	2.70E+07	2.00E+07
Range	0.00E+00	0.00E+00	0.00E+00	0.00E+00	0.00E+00	0.00E+00	1.00E+03	7.10E+06	1.70E+07	1.00E+07

Myricetin

	1000	500	250	125	62.5	0 (V)	*S. mutans*
Minimum	1.00E+02	0.00E+00	1.00E+04	2.90E+05	9.00E+07	6.00E+06	2.40E+08
25% Percentile	1.75E+02	1.25E+02	2.08E+04	2.10E+08	1.40E+08	3.38E+08	2.90E+08
Median	1.05E+03	1.00E+03	3.55E+04	2.85E+08	2.80E+08	4.80E+08	3.20E+08
75% Percentile	3.30E+03	1.68E+03	1.25E+05	3.63E+08	3.80E+08	5.95E+08	3.80E+08
Maximum	3.30E+03	2.70E+03	2.40E+05	4.30E+08	5.60E+08	8.10E+08	4.20E+08
Range	3.20E+03	2.70E+03	2.30E+05	4.30E+08	4.70E+08	8.04E+08	1.80E+08

Compound 1771

	250	125	62.5	31.25	15.625	7.813	3.906	1.953	0.977	0.488	0.244	0 (V)	*S. mutans*
Minimum	0.00E+00	0.00E+00	0.00E+00	0.00E+00	1.00E+02	8.00E+02	1.30E+04	2.00E+04	2.50E+05	1.80E+08	1.80E+08	1.60E+07	5.00E+06
25% Percentile	0.00E+00	7.50E+02	0.00E+00	7.50E+01	1.75E+02	9.50E+02	1.83E+04	2.00E+04	2.50E+05	1.88E+08	1.88E+08	2.13E+07	5.75E+06
Median	0.00E+00	1.50E+03	1.50E+03	5.50E+02	6.00E+02	1.40E+03	2.25E+04	4.00E+04	5.60E+06	2.05E+08	2.05E+08	2.05E+08	7.50E+06
75% Percentile	0.00E+00	5.00E+03	3.25E+03	1.25E+03	8.00E+02	2.20E+03	4.08E+04	5.50E+04	2.55E+08	3.75E+08	3.75E+08	3.88E+08	1.08E+07
Maximum	0.00E+00	8.00E+03	4.00E+03	2.00E+03	8.00E+02	2.20E+03	5.20E+04	7.00E+04	3.00E+08	4.50E+08	4.50E+08	4.50E+08	1.30E+07
Range	0.00E+00	8.00E+03	4.00E+03	2.00E+03	7.00E+02	1.40E+03	3.90E+04	5.00E+04	3.00E+08	2.70E+08	2.70E+08	4.34E+08	8.00E+06

Table S2. Antimicrobial activity of *C. albicans* with compounds: C135, *tt*-farnesol, myricetin, and 1771. Data shown in Figure 2 as CFU/mL.

C135

	1250	1000	500	250	125	62.5	31.25	15.625	0 (V)	*C. albicans*
Minimum	0.00E+00	0.00E+00	0.00E+00	0.00E+00	0.00E+00	0.00E+00	0.00E+00	0.00E+00	1.00E+04	1.20E+07
25% Percentile	0.00E+00	0.00E+00	0.00E+00	0.00E+00	0.00E+00	0.00E+00	0.00E+00	3.20E+03	5.73E+04	1.65E+07
Median	0.00E+00	0.00E+00	0.00E+00	0.00E+00	0.00E+00	0.00E+00	2.00E+02	1.90E+04	1.45E+05	2.05E+07
75% Percentile	0.00E+00	0.00E+00	0.00E+00	0.00E+00	0.00E+00	0.00E+00	5.35E+05	5.75E+04	6.33E+05	2.65E+07
Maximum	0.00E+00	0.00E+00	0.00E+00	0.00E+00	0.00E+00	0.00E+00	7.10E+05	1.20E+05	1.30E+06	3.30E+07
Range	0.00E+00	0.00E+00	0.00E+00	0.00E+00	0.00E+00	0.00E+00	7.10E+05	1.20E+05	1.29E+06	2.10E+07

***tt*-farnesol**

	1250	1000	500	250	125	62.5	31.25	15.625	7.813	0 (V)	*C. albicans*
Minimum	0.00E+00	0.00E+00	0.00E+00	0.00E+00	0.00E+00	1.00E+03	8.00E+03	1.20E+04	1.30E+04	6.00E+04	1.60E+07
25% Percentile	0.00E+00	0.00E+00	0.00E+00	2.00E+02	0.00E+00	3.00E+03	1.60E+04	1.60E+04	2.18E+04	1.00E+06	2.00E+07
Median	0.00E+00	0.00E+00	0.00E+00	6.50E+02	7.00E+02	9.00E+03	1.90E+04	2.30E+04	4.10E+04	6.40E+06	2.35E+07
75% Percentile	0.00E+00	0.00E+00	1.00E+02	1.45E+03	4.63E+03	2.13E+04	2.35E+04	2.93E+04	1.33E+05	1.38E+07	2.88E+07
Maximum	0.00E+00	0.00E+00	2.00E+02	3.00E+03	1.20E+04	2.50E+04	3.00E+04	4.60E+04	5.00E+05	2.90E+07	3.30E+07
Range	0.00E+00	0.00E+00	2.00E+02	3.00E+03	1.20E+04	2.40E+04	2.20E+04	3.40E+04	4.87E+05	2.89E+07	1.70E+07

Myricetin

	1000	500	250	125	0 (V)	*C. albicans*
Minimum	2.10E+03	5.00E+03	1.00E+04	5.00E+03	7.00E+04	1.00E+07
25% Percentile	2.55E+03	1.00E+04	1.50E+04	1.60E+04	1.00E+05	1.40E+07
Median	9.00E+03	1.80E+04	2.00E+04	5.90E+04	4.20E+05	1.85E+07
75% Percentile	1.43E+04	3.00E+04	3.35E+04	2.83E+05	2.83E+06	2.20E+07
Maximum	4.50E+04	5.00E+04	4.20E+04	3.10E+05	5.00E+06	2.90E+07
Range	4.29E+04	4.50E+04	3.20E+04	3.05E+05	4.93E+06	1.90E+07

Compound 1771

	250	125	62.5	31.25	15.625	7.813	3.906	1.953	0.977	0.488	0.244	0 (V)	*C. albicans*
Minimum	7.00E+03	1.10E+05	6.00E+04	4.20E+04	3.20E+04	1.90E+04	8.00E+03	8.00E+03	3.90E+03	3.80E+03	6.00E+03	6.00E+04	1.00E+07
25% Percentile	8.00E+03	1.45E+05	1.05E+05	9.25E+04	3.28E+04	2.15E+04	1.33E+04	1.08E+04	4.48E+03	4.25E+03	1.60E+04	1.43E+05	1.35E+07
Median	1.00E+04	1.75E+05	1.60E+05	1.15E+05	4.00E+04	2.70E+04	1.80E+04	1.30E+04	1.50E+04	1.55E+04	2.20E+04	3.00E+05	1.60E+07
75% Percentile	1.38E+04	2.63E+05	2.63E+05	1.90E+05	5.13E+04	3.23E+04	2.00E+04	1.48E+04	1.73E+04	2.15E+04	2.60E+05	9.75E+05	2.08E+07
Maximum	2.70E+04	3.10E+05	4.10E+05	2.40E+05	5.80E+04	3.70E+04	2.60E+04	1.80E+04	2.40E+04	2.40E+04	5.70E+05	2.30E+06	2.50E+07
Range	2.00E+04	2.00E+05	3.50E+05	1.98E+05	2.60E+04	1.80E+04	1.80E+04	1.00E+04	2.01E+04	2.02E+04	5.64E+05	2.24E+06	1.50E+07

Table S3. Antibiofilm activity of *S. mutans* with compounds: C135, *tt*-farnesol, myricetin, and 1771 of 24h biofilm (initial biofilm formation) and pre-formed biofilms (48h). Data shown in Figure 3 as CFU/mL.

C135 (24 h)

	1250	1000	500	250	125	62.5	0 (V)	*S. mutans*
Minimum	1.00E+02	0.00E+00	0.00E+00	0.00E+00	0.00E+00	0.00E+00	1.50E+06	1.30E+06
25% Percentile	8.00E+02	9.25E+02	0.00E+00	0.00E+00	0.00E+00	0.00E+00	2.33E+06	1.98E+06
Median	2.35E+03	4.00E+03	0.00E+00	0.00E+00	0.00E+00	0.00E+00	4.00E+06	2.50E+06
75% Percentile	4.53E+03	3.83E+04	2.70E+03	0.00E+00	0.00E+00	0.00E+00	1.68E+07	4.60E+06
Maximum	1.60E+04	6.00E+04	9.00E+03	0.00E+00	0.00E+00	0.00E+00	2.40E+07	6.40E+06
Range	1.59E+04	6.00E+04	9.00E+03	0.00E+00	0.00E+00	0.00E+00	2.25E+07	5.10E+06

***tt*-farnesol (24 h)**

	1250	1000	500	250	125	62.5	31.25	0 (V)	*S. mutans*
Minimum	0.00E+00	0.00E+00	0.00E+00	0.00E+00	0.00E+00	0.00E+00	0.00E+00	2.00E+06	4.00E+05
25% Percentile	0.00E+00	0.00E+00	0.00E+00	0.00E+00	0.00E+00	0.00E+00	0.00E+00	2.00E+06	5.00E+05
Median	0.00E+00	0.00E+00	0.00E+00	0.00E+00	0.00E+00	0.00E+00	0.00E+00	3.00E+06	1.15E+06
75% Percentile	0.00E+00	0.00E+00	0.00E+00	0.00E+00	0.00E+00	0.00E+00	0.00E+00	7.25E+06	6.80E+06
Maximum	0.00E+00	0.00E+00	0.00E+00	0.00E+00	0.00E+00	0.00E+00	0.00E+00	8.60E+06	8.00E+06
Range	0.00E+00	0.00E+00	0.00E+00	0.00E+00	0.00E+00	0.00E+00	0.00E+00	6.60E+06	7.60E+06

Myricetin (24 h)

	1250	1000	500	250	0 (V)	*S. mutans*
Minimum	0.00E+00	0.00E+00	0.00E+00	0.00E+00	2.20E+06	8.40E+05
25% Percentile	0.00E+00	0.00E+00	0.00E+00	0.00E+00	2.63E+06	1.44E+06
Median	0.00E+00	0.00E+00	0.00E+00	0.00E+00	1.15E+07	1.70E+06
75% Percentile	0.00E+00	0.00E+00	0.00E+00	0.00E+00	2.43E+07	2.03E+06
Maximum	0.00E+00	0.00E+00	0.00E+00	0.00E+00	2.80E+07	2.20E+06
Range	0.00E+00	0.00E+00	0.00E+00	0.00E+00	2.58E+07	1.36E+06

Compound 1771 (24 h)

	250	125	62.5	31.25	15.625	7.813	3.906	1.953	0 (V)	*S. mutans*
Minimum	0.00E+00	0.00E+00	0.00E+00	0.00E+00	0.00E+00	0.00E+00	0.00E+00	0.00E+00	2.00E+03	1.40E+05
25% Percentile	0.00E+00	0.00E+00	0.00E+00	0.00E+00	0.00E+00	0.00E+00	0.00E+00	0.00E+00	2.08E+05	1.83E+05
Median	0.00E+00	0.00E+00	0.00E+00	0.00E+00	0.00E+00	0.00E+00	0.00E+00	0.00E+00	5.70E+05	2.60E+05
75% Percentile	0.00E+00	0.00E+00	0.00E+00	1.50E+02	0.00E+00	0.00E+00	0.00E+00	0.00E+00	8.58E+06	3.50E+05
Maximum	0.00E+00	0.00E+00	0.00E+00	1.30E+03	0.00E+00	0.00E+00	0.00E+00	3.00E+00	2.20E+07	4.50E+05
Range	0.00E+00	0.00E+00	0.00E+00	1.30E+03	0.00E+00	0.00E+00	0.00E+00	3.00E+00	2.20E+07	3.10E+05

C135 (48 h)

	1250	1000	500	250	125	62.5	0 (V)	*S. mutans*
Minimum	7.00E+05	2.00E+05	5.50E+05	1.70E+05	0.00E+00	0.00E+00	2.70E+04	5.00E+04
25% Percentile	2.18E+06	1.25E+06	1.09E+06	4.63E+05	0.00E+00	2.00E+03	7.68E+04	9.48E+04
Median	2.85E+06	1.90E+06	2.95E+06	9.75E+05	0.00E+00	5.50E+03	1.04E+05	1.16E+05
75% Percentile	1.50E+07	3.65E+06	5.05E+06	2.13E+06	0.00E+00	2.03E+04	2.13E+06	3.65E+06
Maximum	2.60E+07	9.00E+06	6.90E+06	5.10E+06	0.00E+00	4.00E+04	3.10E+06	5.30E+06
Range	2.53E+07	8.80E+06	6.35E+06	4.93E+06	0.00E+00	4.00E+04	3.07E+06	5.25E+06

***tt*-farnesol (48 h)**

	1250	1000	500	250	125	62.5	31.25	0 (V)	*S. mutans*
Minimum	0.00E+00	0.00E+00	0.00E+00	0.00E+00	0.00E+00	0.00E+00	0.00E+00	1.30E+05	1.30E+05
25% Percentile	0.00E+00	0.00E+00	0.00E+00	0.00E+00	0.00E+00	0.00E+00	0.00E+00	1.85E+05	1.83E+05
Median	0.00E+00	0.00E+00	0.00E+00	0.00E+00	0.00E+00	0.00E+00	3.00E+02	2.75E+05	3.10E+05
75% Percentile	0.00E+00	0.00E+00	0.00E+00	0.00E+00	0.00E+00	0.00E+00	7.75E+02	1.00E+06	6.15E+05
Maximum	0.00E+00	0.00E+00	0.00E+00	0.00E+00	0.00E+00	0.00E+00	1.10E+03	2.00E+06	2.30E+06
Range	0.00E+00	0.00E+00	0.00E+00	0.00E+00	0.00E+00	0.00E+00	1.10E+03	1.87E+06	2.17E+06

Myricetin (48 h)

	1250	1000	500	250	0 (V)	*S. mutans*
Minimum	2.00E+03	1.80E+03	1.00E+03	2.70E+03	1.50E+04	1.50E+04
25% Percentile	4.83E+03	3.85E+03	1.25E+03	1.98E+04	3.00E+04	2.75E+04
Median	6.00E+03	5.05E+03	3.20E+03	3.10E+04	1.25E+05	1.85E+05
75% Percentile	9.50E+03	8.35E+03	7.00E+03	5.93E+04	1.80E+05	2.30E+05
Maximum	1.40E+04	9.50E+03	1.30E+04	7.00E+04	2.10E+05	2.60E+05
Range	1.20E+04	7.70E+03	1.20E+04	6.73E+04	1.95E+05	2.45E+05

Compound 1771 (48 h)

	250	125	62.5	31.25	15.625	7.813	3.906	1.953	0 (V)	*S. mutans*
Minimum	1.90E+03	1.10E+03	1.30E+03	1.00E+03	7.60E+03	3.20E+03	4.00E+03	8.30E+03	5.60E+04	1.30E+05
25% Percentile	5.58E+03	5.18E+03	1.75E+03	1.50E+03	1.03E+04	1.63E+04	2.10E+04	3.95E+04	8.90E+04	2.03E+05
Median	8.10E+03	1.10E+04	1.23E+04	1.25E+04	3.50E+04	2.90E+04	2.80E+04	6.30E+04	1.52E+05	2.70E+05
75% Percentile	1.38E+04	1.78E+04	2.38E+04	3.00E+04	5.18E+04	5.58E+04	5.00E+04	7.68E+04	2.35E+05	3.68E+05
Maximum	2.70E+04	3.40E+04	3.90E+04	6.00E+04	7.60E+04	1.06E+05	8.00E+04	9.80E+04	3.30E+05	4.80E+05
Range	2.51E+04	3.29E+04	3.77E+04	5.90E+04	6.84E+04	1.03E+05	7.60E+04	8.97E+04	2.74E+05	3.50E+05

Table S4. Antibiofilm activity of *C. albicans* with compounds: C135, *tt*-farnesol, myricetin, and 1771 of 24h biofilm (initial biofilm formation) and pre-formed biofilms (48h). Data shown in Figure 4 as CFU/mL.

C135 (24 h)

	1250	1000	500	250	125	62.5	31.25	15.625	0 (V)	*C. albicans*
Minimum	0.00E+00	0.00E+00	0.00E+00	0.00E+00	0.00E+00	0.00E+00	0.00E+00	0.00E+00	1.00E+04	7.00E+04
25% Percentile	0.00E+00	0.00E+00	0.00E+00	0.00E+00	0.00E+00	0.00E+00	0.00E+00	0.00E+00	1.58E+04	2.40E+05
Median	0.00E+00	0.00E+00	0.00E+00	0.00E+00	0.00E+00	0.00E+00	0.00E+00	0.00E+00	1.05E+05	2.90E+05
75% Percentile	0.00E+00	0.00E+00	0.00E+00	0.00E+00	0.00E+00	0.00E+00	0.00E+00	0.00E+00	1.58E+05	4.85E+05
Maximum	0.00E+00	0.00E+00	0.00E+00	0.00E+00	0.00E+00	0.00E+00	0.00E+00	0.00E+00	2.30E+05	7.40E+05
Range	0.00E+00	0.00E+00	0.00E+00	0.00E+00	0.00E+00	0.00E+00	0.00E+00	0.00E+00	2.20E+05	6.70E+05

***tt*-farnesol (24 h)**

	1250	1000	500	250	125	62.5	31.25	0 (V)	*C. albicans*
Minimum	0.00E+00	0.00E+00	0.00E+00	0.00E+00	0.00E+00	0.00E+00	0.00E+00	1.00E+05	2.40E+05
25% Percentile	0.00E+00	0.00E+00	0.00E+00	0.00E+00	0.00E+00	0.00E+00	0.00E+00	1.03E+05	2.65E+05
Median	0.00E+00	0.00E+00	0.00E+00	0.00E+00	0.00E+00	0.00E+00	0.00E+00	1.25E+05	3.20E+05
75% Percentile	0.00E+00	0.00E+00	0.00E+00	0.00E+00	0.00E+00	0.00E+00	1.00E+02	1.40E+05	4.28E+05
Maximum	0.00E+00	0.00E+00	0.00E+00	0.00E+00	0.00E+00	0.00E+00	1.00E+02	1.80E+05	6.70E+05
Range	0.00E+00	0.00E+00	0.00E+00	0.00E+00	0.00E+00	0.00E+00	1.00E+02	8.00E+04	4.30E+05

Myricetin (24 h)

	1250	1000	500	250	125	0 (V)	*C. albicans*
Minimum	3.30E+03	1.20E+03	1.00E+02	0.00E+00	0.00E+00	1.50E+03	3.70E+04
25% Percentile	4.60E+03	2.25E+03	5.25E+02	2.50E+02	2.50E+02	4.00E+04	1.18E+05
Median	1.50E+04	4.10E+03	1.10E+03	8.50E+02	8.00E+02	1.90E+05	4.00E+05
75% Percentile	4.60E+04	1.85E+04	2.53E+03	2.33E+03	1.45E+03	2.75E+05	1.03E+06
Maximum	1.40E+05	4.40E+04	6.40E+03	4.10E+03	3.80E+03	3.80E+05	1.20E+06
Range	1.37E+05	4.28E+04	6.30E+03	4.10E+03	3.80E+03	3.79E+05	1.16E+06

Compound 1771 (24 h)

	250	125	62.5	31.25	15.625	7.813	3.906	1.953	0 (V)	*C. albicans*
Minimum	0.00E+00	0.00E+00	0.00E+00	0.00E+00	0.00E+00	0.00E+00	0.00E+00	0.00E+00	9.00E+03	1.00E+04
25% Percentile	0.00E+00	0.00E+00	0.00E+00	0.00E+00	0.00E+00	0.00E+00	0.00E+00	2.50E+02	1.33E+04	2.65E+05
Median	0.00E+00	0.00E+00	0.00E+00	0.00E+00	2.00E+02	0.00E+00	1.00E+02	8.50E+02	6.05E+04	3.85E+05
75% Percentile	0.00E+00	1.33E+04	0.00E+00	1.65E+03	7.00E+02	1.25E+03	3.70E+03	2.58E+03	3.98E+05	5.08E+05
Maximum	1.00E+02	1.90E+04	4.00E+02	4.00E+03	1.70E+03	2.60E+03	7.00E+03	4.60E+03	5.20E+05	5.60E+05
Range	1.00E+02	1.90E+04	4.00E+02	4.00E+03	1.70E+03	2.60E+03	7.00E+03	4.60E+03	5.11E+05	5.50E+05

C135 (48 h)

	1250	1000	500	250	125	62.5	31.25	15.625	0 (V)	*C. albicans*
Minimum	0.00E+00	0.00E+00	0.00E+00	0.00E+00	0.00E+00	0.00E+00	5.00E+02	2.20E+06	2.10E+06	3.30E+06
25% Percentile	0.00E+00	0.00E+00	0.00E+00	0.00E+00	0.00E+00	0.00E+00	1.90E+04	2.35E+06	2.68E+06	3.70E+06
Median	0.00E+00	0.00E+00	0.00E+00	0.00E+00	0.00E+00	0.00E+00	1.30E+06	2.55E+06	3.10E+06	4.10E+06
75% Percentile	0.00E+00	1.00E+02	0.00E+00	2.50E+01	0.00E+00	3.38E+03	2.00E+06	2.85E+06	3.35E+06	4.75E+06
Maximum	0.00E+00	2.00E+02	0.00E+00	8.00E+02	0.00E+00	3.10E+04	2.40E+06	3.30E+06	4.70E+06	5.80E+06
Range	0.00E+00	2.00E+02	0.00E+00	8.00E+02	0.00E+00	3.10E+04	2.40E+06	1.10E+06	2.60E+06	2.50E+06

***tt*-farnesol (48 h)**

	1250	1000	500	250	125	62.5	31.25	0 (V)	*C. albicans*
Minimum	0.00E+00	0.00E+00	1.20E+03	1.00E+02	0.00E+00	1.20E+05	1.00E+06	1.80E+06	2.90E+06
25% Percentile	1.05E+04	6.00E+03	3.93E+03	4.40E+03	2.00E+05	3.33E+05	1.58E+06	3.18E+06	3.50E+06
Median	3.65E+05	2.00E+04	3.00E+04	1.45E+04	3.00E+05	1.15E+06	2.05E+06	3.70E+06	4.00E+06
75% Percentile	1.73E+06	2.95E+05	1.20E+05	2.60E+04	1.10E+06	1.95E+06	3.18E+06	4.45E+06	4.85E+06
Maximum	2.90E+06	8.00E+05	2.90E+05	4.20E+04	1.80E+06	2.50E+06	3.90E+06	6.00E+06	5.60E+06
Range	2.90E+06	8.00E+05	2.89E+05	4.19E+04	1.80E+06	2.38E+06	2.90E+06	4.20E+06	2.70E+06

Myricetin (48 h)

	1250	1000	500	250	125	0 (V)	*C. albicans*
Minimum	1.90E+06	2.20E+06	2.40E+06	2.20E+06	2.30E+06	1.60E+06	2.30E+06
25% Percentile	2.63E+06	3.03E+06	2.93E+06	2.85E+06	2.75E+06	2.18E+06	3.40E+06
Median	2.90E+06	3.35E+06	3.35E+06	3.10E+06	3.10E+06	2.60E+06	3.50E+06
75% Percentile	3.08E+06	3.93E+06	4.20E+06	3.58E+06	3.28E+06	2.78E+06	3.95E+06
Maximum	4.60E+06	4.60E+06	4.90E+06	4.20E+06	3.40E+06	3.50E+06	4.10E+06
Range	2.70E+06	2.40E+06	2.50E+06	2.00E+06	1.10E+06	1.90E+06	1.80E+06

Compound 1771 (48 h)

	250	125	62.5	31.25	15.625	7.813	3.906	1.953	0 (V)	*C. albicans*
Minimum	0.00E+00	1.20E+06	9.00E+03	3.20E+04	1.10E+05	1.90E+06	1.50E+06	1.70E+05	1.10E+06	2.70E+06
25% Percentile	0.00E+00	2.00E+06	4.25E+05	5.40E+04	1.55E+06	2.00E+06	2.45E+06	8.35E+05	1.73E+06	3.48E+06
Median	3.20E+04	2.30E+06	1.60E+06	1.50E+06	2.70E+06	2.35E+06	3.40E+06	3.15E+06	2.15E+06	4.50E+06
75% Percentile	5.73E+05	2.68E+06	2.13E+06	2.48E+06	4.00E+06	2.88E+06	3.73E+06	4.43E+06	3.38E+06	5.83E+06
Maximum	1.40E+06	3.20E+06	2.90E+06	3.10E+06	5.00E+06	3.60E+06	5.30E+06	5.70E+06	5.10E+06	7.40E+06
Range	1.40E+06	2.00E+06	2.89E+06	3.07E+06	4.89E+06	1.70E+06	3.80E+06	5.53E+06	4.00E+06	4.70E+06

Table S5. Antibiofilm activity of dual-species *S. mutans* and *C. albicans* biofilms with combined compounds (with and without sodium fluoride): C135 (C), *tt*-farnesol (Far), myricetin (Myr), 1771, and sodium fluoride (F). Data shown in Figure 5 as CFU/mL.

***S. mutans* (24 h)**

	C	C+Far	C+1771	C+Mir	C+Far+1771	C+Far+Mir	C+Far+1771+Mir	C+1771+Mir	Far+1771	1771+Mir	Far+1771+Mir	C+F	C+Far+F	C+1771+F	C+Mir+F	C+Far+1771+Mir+F	0 (V)	*S. mutans*
Minimum	0.00E+00	0.00E+00	0.00E+00	0.00E+00	0.00E+00	0.00E+00	0.00E+00	0.00E+00	0.00E+00	0.00E+00	0.00E+00	0.00E+00	0.00E+00	0.00E+00	0.00E+00	0.00E+00	3.40E+05	3.40E+06
25% Percentile	0.00E+00	0.00E+00	0.00E+00	0.00E+00	0.00E+00	0.00E+00	0.00E+00	0.00E+00	0.00E+00	0.00E+00	0.00E+00	0.00E+00	0.00E+00	0.00E+00	0.00E+00	0.00E+00	4.43E+06	4.00E+06
Median	0.00E+00	0.00E+00	0.00E+00	0.00E+00	0.00E+00	0.00E+00	0.00E+00	0.00E+00	0.00E+00	0.00E+00	0.00E+00	0.00E+00	0.00E+00	0.00E+00	0.00E+00	0.00E+00	5.75E+06	4.90E+06
75% Percentile	0.00E+00	0.00E+00	0.00E+00	0.00E+00	0.00E+00	0.00E+00	0.00E+00	0.00E+00	0.00E+00	0.00E+00	0.00E+00	0.00E+00	0.00E+00	0.00E+00	0.00E+00	0.00E+00	1.16E+07	5.53E+06
Maximum	0.00E+00	0.00E+00	0.00E+00	0.00E+00	0.00E+00	0.00E+00	0.00E+00	0.00E+00	0.00E+00	0.00E+00	0.00E+00	0.00E+00	0.00E+00	0.00E+00	0.00E+00	0.00E+00	1.40E+07	5.90E+06
Range	0.00E+00	0.00E+00	0.00E+00	0.00E+00	0.00E+00	0.00E+00	0.00E+00	0.00E+00	0.00E+00	0.00E+00	0.00E+00	0.00E+00	0.00E+00	0.00E+00	0.00E+00	0.00E+00	1.37E+07	2.50E+06

***S. mutans* (48 h)**

	C	C+Far	C+1771	C+Mir	C+Far+1771	C+Far+Mir	C+Far+1771+Mir	C+1771+Mir	Far+1771	1771+Mir	Far+1771+Mir	C+F	C+Far+F	C+1771+F	C+Mir+F	C+Far+1771+Mir+F	0 (V)	*S. mutans*
Minimum	0.00E+00	0.00E+00	0.00E+00	0.00E+00	0.00E+00	0.00E+00	0.00E+00	0.00E+00	0.00E+00	1.00E+05	0.00E+00	0.00E+00	0.00E+00	0.00E+00	0.00E+00	0.00E+00	2.20E+06	1.00E+07
25% Percentile	1.88E+03	0.00E+00	1.33E+03	1.25E+02	0.00E+00	0.00E+00	0.00E+00	7.50E+01	4.75E+03	4.75E+05	0.00E+00	0.00E+00	0.00E+00	0.00E+00	0.00E+00	0.00E+00	3.33E+06	1.60E+07
Median	7.95E+03	0.00E+00	2.15E+03	2.50E+02	0.00E+00	0.00E+00	0.00E+00	2.50E+03	2.10E+04	1.90E+06	2.00E+04	0.00E+00	0.00E+00	0.00E+00	0.00E+00	0.00E+00	3.85E+06	1.95E+07
75% Percentile	2.25E+04	1.50E+02	6.78E+03	1.28E+04	7.50E+01	0.00E+00	0.00E+00	1.03E+04	9.00E+04	9.13E+06	4.00E+04	0.00E+00	0.00E+00	0.00E+00	0.00E+00	0.00E+00	1.00E+07	2.20E+07
Maximum	5.20E+04	2.00E+02	1.30E+04	2.30E+04	2.00E+02	0.00E+00	0.00E+00	2.00E+04	1.30E+05	1.10E+07	1.00E+05	0.00E+00	0.00E+00	0.00E+00	0.00E+00	0.00E+00	1.70E+07	3.60E+07
Range	5.20E+04	2.00E+02	1.30E+04	2.30E+04	2.00E+02	0.00E+00	0.00E+00	2.00E+04	1.30E+05	1.09E+07	1.00E+05	0.00E+00	0.00E+00	0.00E+00	0.00E+00	0.00E+00	1.48E+07	2.60E+07

***C. albicans* (24 h)**

	C	C+Far	C+1771	C+Mir	C+Far+1771	C+Far+Mir	C+Far+1771+Mir	C+1771+Mir	Far+1771	1771+Mir	Far+1771+Mir	C+F	C+Far+F	C+1771+F	C+Mir+F	C+Far+1771+Mir+F	0 (V)	*C. albicans*
Minimum	0.00E+00	0.00E+00	0.00E+00	0.00E+00	0.00E+00	0.00E+00	0.00E+00	0.00E+00	0.00E+00	0.00E+00	0.00E+00	0.00E+00	0.00E+00	0.00E+00	0.00E+00	0.00E+00	2.20E+04	5.00E+05
25% Percentile	0.00E+00	0.00E+00	0.00E+00	0.00E+00	0.00E+00	0.00E+00	0.00E+00	0.00E+00	0.00E+00	0.00E+00	0.00E+00	0.00E+00	0.00E+00	0.00E+00	0.00E+00	0.00E+00	1.85E+05	1.83E+06
Median	0.00E+00	0.00E+00	0.00E+00	0.00E+00	0.00E+00	0.00E+00	0.00E+00	0.00E+00	0.00E+00	0.00E+00	0.00E+00	0.00E+00	0.00E+00	0.00E+00	0.00E+00	0.00E+00	2.90E+05	2.05E+06
75% Percentile	0.00E+00	0.00E+00	0.00E+00	0.00E+00	0.00E+00	0.00E+00	0.00E+00	0.00E+00	0.00E+00	0.00E+00	0.00E+00	0.00E+00	0.00E+00	0.00E+00	0.00E+00	0.00E+00	3.95E+05	2.50E+06
Maximum	0.00E+00	0.00E+00	0.00E+00	0.00E+00	0.00E+00	0.00E+00	0.00E+00	0.00E+00	0.00E+00	0.00E+00	0.00E+00	0.00E+00	0.00E+00	0.00E+00	0.00E+00	0.00E+00	1.20E+06	3.40E+06
Range	0.00E+00	0.00E+00	0.00E+00	0.00E+00	0.00E+00	0.00E+00	0.00E+00	0.00E+00	0.00E+00	0.00E+00	0.00E+00	0.00E+00	0.00E+00	0.00E+00	0.00E+00	0.00E+00	1.18E+06	2.90E+06

***C. albicans* (48 h)**

	C	C+Far	C+1771	C+Mir	C+Far+1771	C+Far+Mir	C+Far+1771+Mir	C+1771+Mir	Far+1771	1771+Mir	Far+1771+Mir	C+F	C+Far+F	C+1771+F	C+Mir+F	C+Far+1771+Mir+F	0 (V)	*C. albicans*
Minimum	0.00E+00	0.00E+00	0.00E+00	0.00E+00	0.00E+00	0.00E+00	0.00E+00	0.00E+00	6.00E+03	4.00E+05	5.00E+05	0.00E+00	0.00E+00	0.00E+00	0.00E+00	0.00E+00	1.70E+06	3.20E+06
25% Percentile	0.00E+00	0.00E+00	0.00E+00	0.00E+00	0.00E+00	0.00E+00	0.00E+00	3.50E+02	7.28E+04	1.38E+06	9.00E+05	0.00E+00	0.00E+00	1.75E+02	0.00E+00	0.00E+00	2.53E+06	3.50E+06
Median	0.00E+00	0.00E+00	0.00E+00	0.00E+00	0.00E+00	0.00E+00	0.00E+00	1.35E+03	2.25E+05	1.90E+06	1.15E+06	0.00E+00	0.00E+00	4.05E+03	0.00E+00	0.00E+00	3.25E+06	4.00E+06
75% Percentile	0.00E+00	0.00E+00	0.00E+00	0.00E+00	4.43E+03	0.00E+00	0.00E+00	3.63E+03	2.90E+05	2.23E+06	1.43E+06	1.30E+03	0.00E+00	4.78E+04	4.75E+02	0.00E+00	3.75E+06	4.48E+06
Maximum	0.00E+00	0.00E+00	0.00E+00	0.00E+00	8.40E+03	0.00E+00	2.00E+02	7.10E+03	4.10E+05	4.90E+06	2.10E+06	3.00E+04	0.00E+00	6.10E+04	1.40E+03	0.00E+00	4.50E+06	5.20E+06
Range	0.00E+00	0.00E+00	0.00E+00	0.00E+00	8.40E+03	0.00E+00	2.00E+02	7.10E+03	4.04E+05	4.50E+06	1.60E+06	3.00E+04	0.00E+00	6.10E+04	1.40E+03	0.00E+00	2.80E+06	2.00E+06
